# Rheological Properties and Physical Stability of Aqueous Dispersions of Flaxseed Fibers

**DOI:** 10.3390/gels10120787

**Published:** 2024-12-02

**Authors:** María-Carmen Alfaro-Rodríguez, María Carmen García, Paula Prieto-Vargas, José Muñoz

**Affiliations:** Departamento de Ingeniería Química, Escuela Politécnica Superior, Universidad de Sevilla, E41011 Sevilla, Spain; mcgarcia@us.es (M.C.G.); paulaprietovargas@gmail.com (P.P.-V.); jmunoz@us.es (J.M.)

**Keywords:** flaxseed fiber, weak gel, viscoelasticity, flow, stability, homogenization rate

## Abstract

The main objective of this work is to investigate the influence of shear on the rheological properties and physical stability of aqueous dispersions of flaxseed fiber. The variable to consider will be the homogenization rate in two different rotor-stator homogenizers, Ultraturrax T50 or T25. In order to achieve the proposed objective, small amplitude oscillatory tests, flow curves, and multiple light scattering measurements were carried out. All samples exhibited a shear thinning behavior that was not influenced by the shear imposed, and a weak gel-like behavior. The latter, unlike the flow behavior, was sensitive to the homogenization rate. Thus, an increase in this variable caused a decrease in the viscoelastic moduli values. This result pointed out a weakening of the network formed by the flaxseed fiber in an aqueous medium. On the contrary, the physical stability improved. Nevertheless, all samples were highly stable. The homogenizer used was a significant variable. The shear negatively influenced the microstructure of the aqueous flaxseed fiber dispersions, although the obtained gels were highly stable. The gel-like behavior, the high viscosity at low shear rates, and the high physical stability of the samples studied make them interesting food stabilizers and thickeners.

## 1. Introduction

Today, there is a tendency to care for food habits to achieve a positive impact on health. In this sense, it has produced an increase in the consumption of natural ingredients with high nutritional value instead of artificial food additives. An interesting option to fulfill this purpose is the use of dietary fibers in food formulation [1]. Their health advantages include promoting normal laxation, the regulation of intestinal transit, preventing or treating colon cancer or cardiovascular diseases, and reducing blood cholesterol levels [2].

With this objective, in this work, the flaxseed fiber obtained from flax (*Linum usitatissimum* L.) has been studied to include it in the diet. Different studies have demonstrated that flaxseed fiber is potentially related to a lower incidence of prevalent diseases in today’s society such as obesity, diabetes, cancer, or heart diseases [3,4]. Flaxseed fiber is a mainly soluble dietary fiber, which is found in the outermost layer of the flax hull. In fact, it is a by-product of the flax oil industry [4]. A mucilaginous material is liberated when seeds are wetted in water, resulting in soluble flaxseed gum [5]. The extraction yield, the rheological and physicochemical properties, and the amount of protein of the fiber depend on the temperature, pH, extraction time, the ratio of water/seeds, and the variety of the raw material [6,7]. The structure of flaxseed fiber has been widely studied [5,6,8]. Flaxseed fiber has a highly branched polymeric structure of high complexity, consisting of polysaccharide fractions with various molecular weights composed of monosaccharides [9]. The nature of the monosaccharide and its abundance is variable and it determines the properties of the fiber. The main monosaccharides consist of L-galactose, D-xylose, L-rhamnose, and D-galacturic acid, and, to a lesser extent, other constituents such as L-arabinose, D-glucose, and L-fucose [10]. Depending on the proportion of these monomers, this fiber can be classified as more neutral, if contains a β-D-(1→4) xylan backbone of arabinoxylan with L-arabinose and D-galactose, or more acidic, if it contains L-rhamnose, D-galactose, and D-glucuronic acid [4,10,11]. Due to its structure, flaxseed fiber acts as a hydrocolloid.

Hydrocolloids or gums are molecules of high molecular weight consisting of long chains of hydrophilic substances [12], which make them have a high affinity for water and tend to bind to it. For this reason, hydrocolloids in aqueous systems produce highly viscous dispersions or gels [13]. Hydrocolloids can be natural, synthetic, or semi-synthetic. However, today, the consumer prefers products containing natural ingredients. Natural gums can be classified according to their origin as plant exudates (tree or shrub exudates), plant seeds, seaweed extracts, microorganisms (bacteria) and animal sources. Among them, plant-based hydrocolloids, such as flaxseed fiber, are better regarded by consumers than those of animal origin. These hydrocolloids are widely used in the food industry due to not only their multiple environmental benefits (biodegradable, renewable, nontoxic, obtainable, etc.) but especially their numerous functional properties [12]. This fact is a consequence of their ability to modify the rheological properties of the formulation, especially flow behavior and mechanical properties, related to the viscosity and texture of food systems, respectively [14]. For these reasons, hydrocolloids are extensively used as emulsifying, stabilizing, thickening, and gelling agents. Hydrocolloid aqueous solutions are used to form gels, that is, materials with a midway behavior between a solid and a liquid, exhibiting mechanical rigidity and showing flow capacity [15]. However, from the rheological point of view, the gel is a viscoelastic material, with the elastic component (G′) higher than the viscous component (G″) [16]. Additionally, it is possible to distinguish between weak gels and strong gels by carrying out non-destructive measurements such as dynamic frequency tests at constant shear stress within the linear viscoelastic region previously obtained. In the case of flaxseed fiber, gel formation in aqueous solution is due to the presence of arabinoxylan in its composition. It typically occurs through covalent cross-links formed between the ferulic acid residues of the adjacent α-l-Ara f side chain [17]. As Wang et al. (2025) [18] reported, a slight increase in molecular mass produces a moderate increase in aggregation state and apparent viscosity. Furthermore, the rheological properties are directly related to the molecular weight of the arabinose/xylan ratio and the degree of branching [18,19].

Flaxseed fiber, as a hydrocolloid, has techno-functional properties such as structure formation properties, gelling capacity, interfacial and surface properties, or oil- or water-holding capacity [20,21,22,23]. Consequently, this fiber, used years ago in cosmetics [7], can be easily included in dairy products and beverages [20,21,24,25]. Additionally, it can be used as a possible fiber fortifier in foods [5]. Nevertheless, the functional properties mentioned above can be modified by the mechanical processes usually used to obtain the food system, and therefore the rheological properties and physical stability of these systems can also be changed. For this reason, an understanding of these properties as a function of shear is necessary. The literature provides information about the flow and viscoelastic behavior of aqueous systems of flaxseed gum as a function of temperature, salt, or pH compared to other gums [26,27]. However, there is a lack of a comprehensive study of the effect of the energy applied during the preparation of aqueous flaxseed fiber dispersions on the properties mentioned above.

In this work, two rotor-stator homogenizers, Ultraturrax T25 and Ultraturrax T50, were used at different homogenization rates for the dispersion of a plant-based gum, such as flaxseed fiber, in water. The aim of this research and, at the same time, the novelty, was to know the effect of the energy applied during the homogenization of the fiber dispersions on the physical stability, the flow behavior, viscoelastic properties, and microstructure of these dispersions. Flow curves, small amplitude oscillatory tests, multiple light scattering tests, and optical microscopy were carried out to know the effect of shear and evaluate the potential use of soluble flaxseed fiber in the food formulation.

The results of this study could be useful for the design and development of functional oil-in-water emulsions. Microstructural changes caused by shear may occur in the fiber that could provoke variations in rheological properties. Thus, controlling the consumption of energy, it could be possible to design a flaxseed fiber aqueous dispersion with desirable rheological properties to be used as a food emulsion stabilizer or thickener.

## 2. Results and Discussion

### 2.1. Energy Consumption

Table 1 shows the values of the consumption of energy per unit mass (E) of different aqueous dispersions of flaxseed fiber calculated from Equation (1):

E = (P·t)/M,(1)

where P is the consumption of electrical power, t is the homogenization time, and M is the mass of the sample. As can be observed, and as expected, an increase in the homogenization rate caused an increase in the E values regardless of the rotor-stator device used. Interestingly, if we compare the energy consumption per unit mass of the Ultraturrax T50 and T25 devices at 6000 and 6500 rpm, respectively, a higher value is observed in the latter, which could be explained by considering not only the different geometry of the rotor [28], but also the volume of sample treated.

### 2.2. Stress Sweep

**Figure 1:**
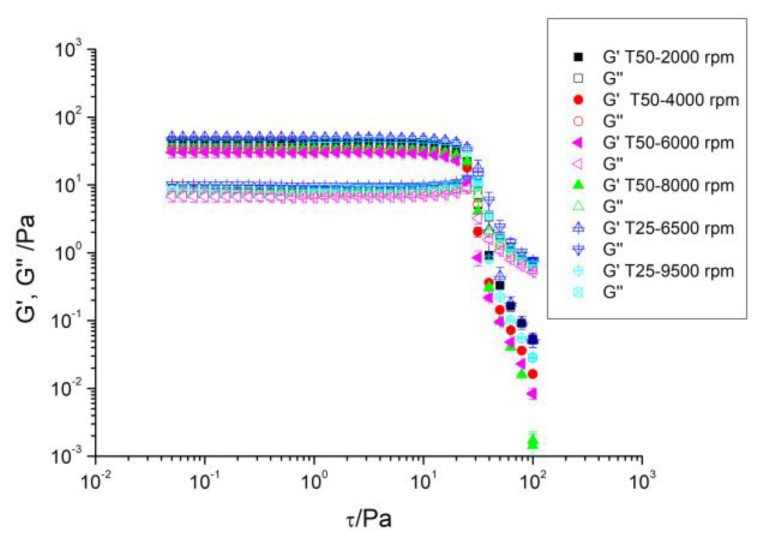
Linear viscoelastic region of aqueous dispersions of flaxseed fiber as a function of homogenization rate and geometry.

Figure 1 shows the stress amplitude sweeps performed from 0.05 Pa to 100 Pa at a frequency of 1 Hz for all samples investigated. Two zones can be distinguished: (a) a zone in which the viscoelastic modules, the storage modulus (G′, a measure of the elasticity), and the loss modulus (G″, representing the viscous component) remain practically constant, and (b) a zone in which the values of both decrease. Actually, for G″, and before this decrease in its values occurs, a pseudodilatant peak takes place. This behavior has also been found in some gums and emulsions [29,30,31] and has been attributed to a microstructure rearrangement at these high stresses. Afterwards, once a certain stress value is exceeded, the sample flows. The stress from which one of the moduli does not remain constant is known as critical stress (τ_c_) and its value determines the extension of the linear viscoelastic region (LVR). The value of this stress, also known as yield stress, gives an idea of the degree of association that exists between macromolecules [30] and is a measure of the ease of breaking the gel. Thus, a low value means that the force required to break the gel is small, and therefore it is easily broken. Interestingly, the values of τ_c_ for the samples studied turned out to be similar (around 10 Pa) and therefore independent of the homogenization rate and geometry used during the preparation of the dispersion. In short, the homogenization rate and geometry had a negligible effect on the extension of the LVR. Similar results were found by fiber dispersions of apple, carrot, and potato pulp [32]. On the other hand, Figure 1 evidenced that all samples exhibited a predominant elastic behavior within the linear viscoelastic region by showing G′ values above those of G″.

### 2.3. Frequency Sweep

**Figure 2:**
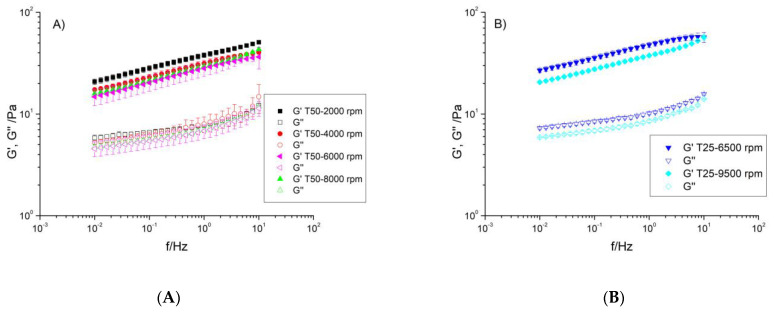
Mechanical spectra of aqueous dispersions of flaxseed fiber as a function of homogenization rate in (**A**) the Ultraturrax T50 device and (**B**) the Ultraturrax T25 device. T = 20 °C.

Dynamic frequency sweep tests were performed at a stress located within the LVR to know the dependence on the frequency of G′ and G″. This nondestructive test is very useful to investigate the basic nature of these dispersions and will allow aqueous fiber dispersions to be classified into one of the following four groups: (a) dilute solutions, (b) crosslinking or concentrated solutions, (c) weak gels, and (d) strong gels [33,34]. Figure 2 illustrates the mechanical spectra of the aqueous dispersions of flaxseed fiber as a function of homogenization rate and geometry. All samples exhibited the same mechanical behavior. Thus, G′ values were greater than those of G″ in the whole range of frequencies studied, indicating a gel-like structure, but their magnitude was less than 10 times that of G″ [35,36]. Furthermore, there was a small dependence on frequency [33,37,38]. Therefore, from a rheological point of view, these systems behaved as weak gels. This result is in agreement with Yu et al. (2017) [17] and Liu et al. (2018) [4] and was attributed to the high arabinoxylan content. It should be noted that the rheological behavior and structure of flaxseed fiber can vary depending on several factors such as the extraction method, the origin of the raw material, the processing conditions of the dispersions, etc. By the way, Chang et al. (2017) [27] published small amplitude oscillatory shear results for flaxseed gum at 2 wt% and they also found that the system behaved as a solid with storage modulus, G′, higher than loss modulus, G″. However, the values of both moduli were clearly greater than those obtained in this research and the frequency dependence was lower.

**Figure 3:**
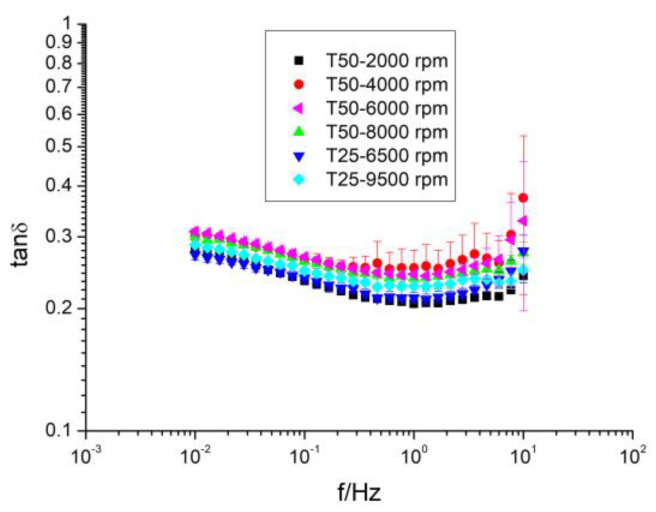
Evolution of tanδ with frequency of aqueous dispersions of flaxseed fiber as a function of homogenization rate and geometry. T = 20 °C.

The viscoelastic behavior of the samples can also be analyzed by the loss tangent, tanδ (Figure 3), which represents the relationship between the energy lost per cycle and the energy stored per cycle (G″/G′). Thus, when tanδ < 1 it means that elastic behavior predominates, while a value of tanδ > 1 means that viscous behavior predominates. In polymeric systems, the literature provides the following numerical ranges for tanδ: (a) very high values for dilute solutions, (b) 0.2 < tanδ < 0.3 for amorphous polymers, and (c) low values, close to 0.01, for crystalline polymers and gels [34]. As can be observed in Figure 3, the values of tanδ were lower than one in all of the frequency ranges, pointing out that these dispersions were more elastic than viscous. Their values were higher than 0.1 and ranged from 0.24 to 0.30, which was typical of the so-called weak gels [38]. In addition, a slight dependence on frequency was found. These samples exhibited a decrease in tanδ values at low frequencies (for high observation times), while remaining practically constant for short observation times. Actually, a very slight increase was observed but this variation was not significant. A similar behavior was found by Chang et al. (2017) [27], but in that work the values of the loss tangent were slightly higher, which can be explained as indicated above (different conditions can offer different microstructures and, therefore, different rheological behaviors). Other hydrocolloid aqueous dispersions, such as diutan or rhamsan gums (microbial gums), sterculia apetala or sterculia striata gums (exudate gums) [31,39,40], and guar, locust bean, or tamarind gums (plant-based gums) [27], also exhibited an elastic response higher than the viscous response and, therefore, values of tanδ below one.

**Figure 4:**
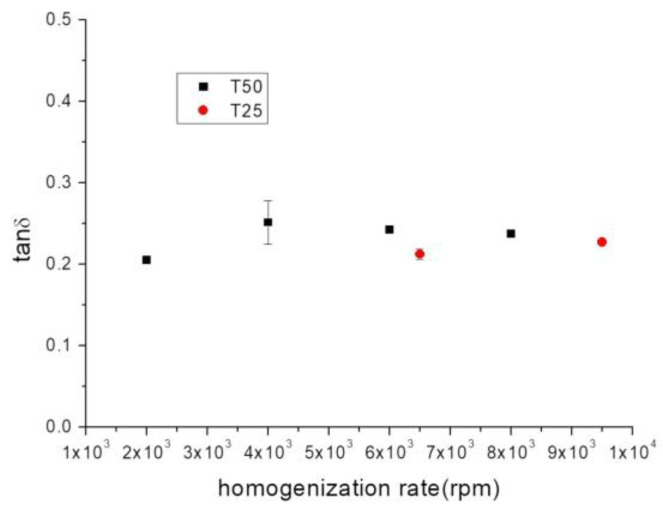
Tanδ, at a fixed frequency of 1 Hz, versus the homogenization rate of aqueous dispersions of flaxseed fiber as a function of the homogenizer geometry. T = 20 °C.

Regardless of the equipment used, an increase in the homogenization rate caused a decrease in the values of the viscoelastic moduli. This result points to a weakening of the network formed by the flaxseed fiber in solution and, therefore, a negative effect of shear on the microstructure formed by the fiber. Further analysis can be performed by analyzing the loss tangent values at a fixed frequency of 1 Hz (Figure 4). At a fixed geometry, the lowest values were presented for the sample submitted to the lowest homogenization rate, that is, T50-2000 rpm and T25-6500 rpm, respectively. This result, as abovementioned, was due to the occurrence of a stronger structure with higher relaxation times. It is well known that a strong network leads to a product that is better able to prevent particle sedimentation than a weak network. From this point of view, the lower application of shear led to the formation of a gel-like structure with better stabilizing properties. García et al. (2017) [41] also studied the influence of shear on the rheological properties of aqueous diutan gum dispersions but, unlike our research, they applied microfluidization. In that work, the high shear applied, much higher than that provided to our samples, caused a change in behavior from predominantly elastic to predominantly viscous that was attributed to the decrease in the molecular weight and the increase in the polydispersity, and consequently, a high loss of the structured network that the macromolecule formed in solution.

It is worth noting that the energy consumption per mass unit is higher in the Ultraturrax T25 rotor-stator homogenizer (compare 6000 rpm and 6500 rpm), but the weakening of the network is not higher (compare 8000 rpm and 9500 rpm). Even more evident is the fact that at 9500 rpm in T25, where the greatest energy per unit of mass is applied, the sample presents similar values of G′ and G″ to those of 2000 rpm in T50 (the sample with the lowest energy applied). This result suggests that the efficiency of the T25 device is lower than that of the T50.

### 2.4. Flow Curves

**Figure 5:**
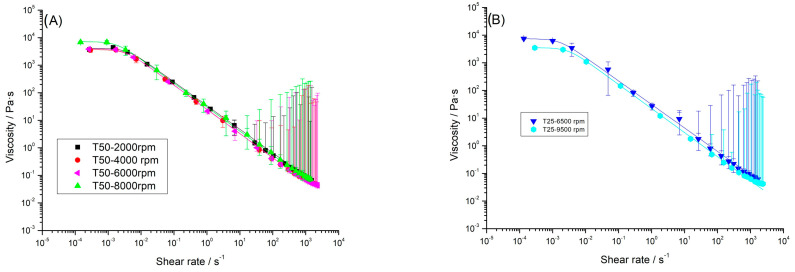
Influence of shear rate on the apparent viscosity of aqueous dispersions of flaxseed fiber as a function of the homogenization rate in (**A**) the Ultraturrax T50 device and (**B**) the Ultraturrax T25 device. T = 20 °C.

The influence of the shear rate on the viscosity of the aqueous dispersions of flaxseed fiber is shown in Figure 5. The samples flowed as expected for a weak gel, and they exhibited a shear thinning behavior with an incipient Newtonian region at low shear rates. Other authors also found pseudoplastic flow behavior for this fiber [42] and other gum or fiber dispersions such as those obtained from Kinnow mandarine [43], guar gum, cress seed gum, cassia gum, tamarind gum, or basil see gum [26,27]. This behavior could be attributed to the high molecular mass of arabinoxylan present in the fiber [4] and may be explained by considering a higher disruption rate than the formation rate of the entanglements between molecules by increasing the shear rate.

Different flow models have been used in literature to fit the experimental results of flow curves for aqueous hydrocolloid or gum dispersions such as Cross, Carreau, Morris or power law models [26,44]. In this work, the results were well fitted to the Carreau model [45], as can be deduced from the high values of the regression coefficient R^2^, which was greater than 0.99 in all cases, using Equation (2).

(2) $$\eta_{=}\frac{\eta_{0}}{{\left(1+{\left(\frac{\gamma }{\dot{\gamma_{c}}}\right)}^{2}\right)}^{\left[\frac{1-n}{2}\right]}}$$

where *η* is the apparent viscosity, *η*_0_ is the apparent viscosity at a low shear rate, $\dot{\gamma_{c}}$ and $\dot{\gamma }$ are the critical shear rate associated with the onset of structural collapse and the shear rate, respectively, and n is the flow behavior index. This parameter has a value of one for a Newtonian sample and decreases to zero with an increase in shear thinning behavior. It should be noted that the Newtonian region at high shear rate (*η_∞_*) was not achieved in these samples, and for this reason, the Carreau model presented is a simplified version.

The fitting parameters for the Carreau model are shown in Table 2. The flow curves turned out to be insensitive to the homogenization rate and geometry, showing values of apparent viscosity with the same order of magnitude. No trend was found in either the critical shear rate or the flow index. Furthermore, the flow index values had values close to 0.13, which pointed out a marked shear thinning and predominantly solid behavior. The importance of this fact must be highlighted since, in view of its application as a thickener, a low value for this parameter is imperative [46]. Other plant-based gums also exhibited a low flow index value, such as sage seed gum (n = 0.20), basil seed gum (n = 0.17), Balangu-Shirazi seed gum (n = 0.29) [26], or tara gum (n = 0.18) [44], while others, such as locust bean gum (n = 0.31), guar gum (n = 0.47), or tamarind gum (n = 0.50) [27], exhibited clearly higher values.

### 2.5. Microscopy

**Figure 6:**
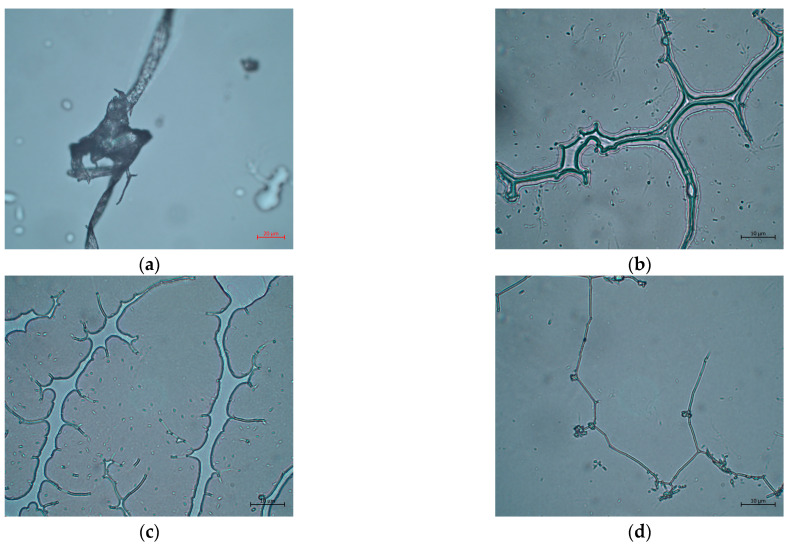
Optical micrographs of aqueous dispersions of flaxseed fiber obtained with (**a**) Ultraturrax T50 at 2000 rpm, objective: 40×; (**b**) Ultraturrax T50 at 8000 rpm, objective: 100×; (**c**) Ultraturrax T25 at 6500 rpm, objective: 100×; and (**d**) Ultraturrax T25 at 9500 rpm, objective: 100×.

From a microstructural point of view, the increase in the homogenization rate caused a decrease in the size of the insoluble components of the fiber, as can be observed in the micrographs shown in Figure 6. The authors hypothesize that this decrease in size should lead to an increase in the exposure area of the fiber and, probably, to a greater water retention capacity, implying an increase in viscosity and viscoelasticity. However, this increase would be countered by the destruction of part of the junction zones that form the basis of the three-dimensional network [14] characteristic of these gels, resulting in a decrease in the viscoelastic modules, G′ and G″.

### 2.6. Physical Stability

**Figure 7:**
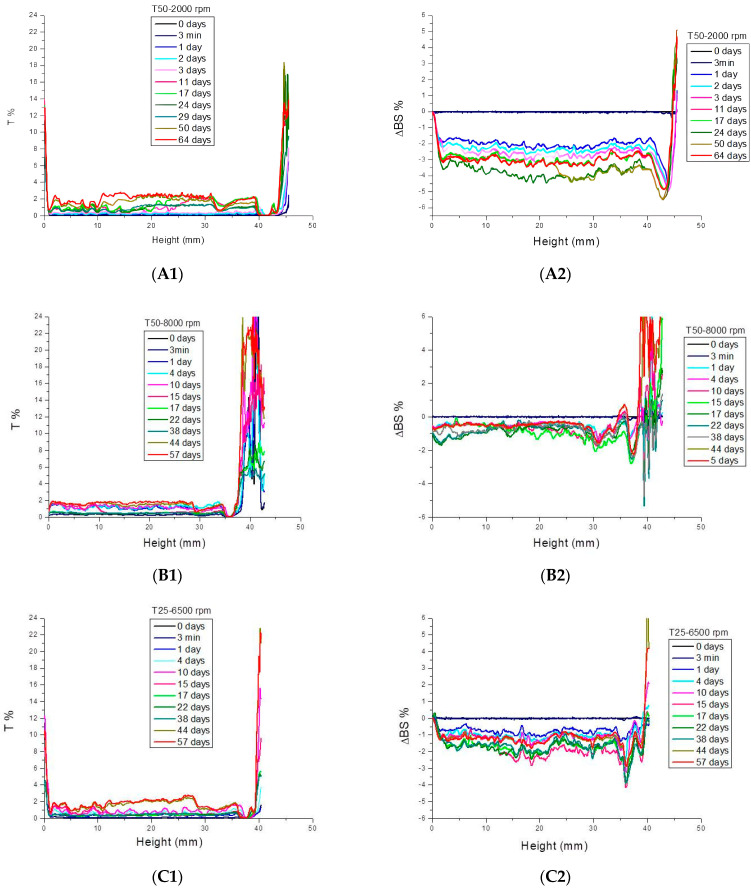
Delta backscattering percentage versus height of the vial containing the aqueous dispersions of flaxseed fiber as a function of the aging time: (**A1**) T50-2000 rpm, (**B1**) T50-8000 rpm, and (**C1**) T25-6500 rpm. Transmittance % versus height of the vial containing the aqueous dispersions of flaxseed fiber as a function of the aging time (**A2**) T50-2000 rpm, (**B2**) T50-8000 rpm, and (**C2**) T25-6500 rpm. T = 20 °C.

**Figure 8:**
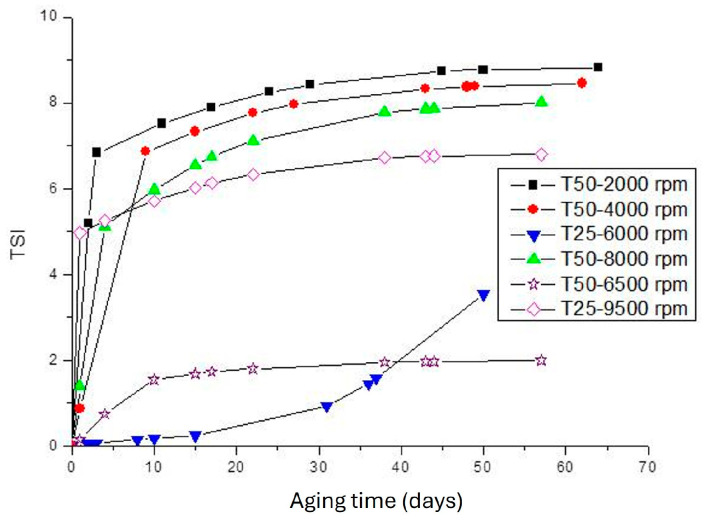
Turbiscan Stability Index of aqueous dispersions of flaxseed fiber versus storage time as a function of the homogenization rate and geometry.

**Figure 9:**
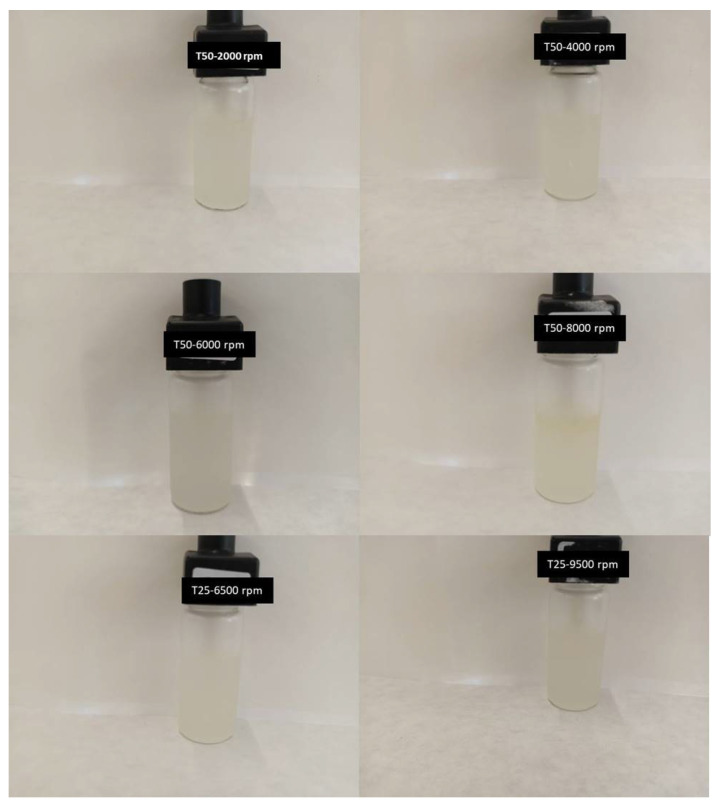
Visual inspection results of aqueous dispersions of flaxseed fiber three months after preparation.

The physical stability of fiber dispersions was studied using a multiple light scattering technique and the delta backscattering and transmittance profiles were analyzed at different aging times. By way of example, Figure 6 illustrates the profiles of T% and ΔBS % for T50-2000 rpm (Figure 7(A1,A2)), T50-8000 rpm (Figure 7(B1,B2)), and T25-6500 rpm (Figure 7(C1,C2)). It should be noted that the main changes occurred during the first 24 h after preparation, but afterwards the profiles only just changed and remained practically motionless, without notable changes. To compare the stability of different samples, the Turbiscan Stability Index (TSI) values were calculated as a function of storage time and plotted in Figure 8. As can be observed, the samples obtained at an intermediate rate value with Ultraturrax T50 or the lowest studied with Ultraturrax T25 presented the lowest TSI value, and therefore the greatest physical stability. However, the high stability of these samples is worth noting since ΔBS % was below 5% [47,48]. In fact, three months after their preparation, they remained visually stable, as can be deduced from the photos shown in Figure 9. The appearance of these gels was that of a non-translucent whitish gel. In addition, again, as occurred with viscoelastic moduli of mechanical spectra, it should be noted that there is no direct relationship between physical stability and applied energy when different geometries are involved.

## 3. Conclusions

Flaxseed fiber gels have been developed. Shear negatively influenced the weak gel-like structure obtained by submitting the aqueous fiber dispersions to rotor-stator treatment, causing a weakening of the three-dimensional network attained at the lowest homogenization rates. Thus, with higher applied energy, lower values of viscoelastic moduli can be reached. However, this result was also dependent on the geometry used since higher energy applied in different geometry did not cause the expected modification. The results showed a lower efficiency of the Ultraturrax T25 device, that is, part of the energy applied in this device was lost, probably due to friction. The sample obtained with Ultraturrax T50 at 2000 rpm and with Ultraturrax T25 at 6500 rpm exhibited the best viscoelastic properties in view of its potential application as a stabilizer. All samples flowed, and they exhibited shear thinning flow behavior with very high viscosity at low shear rates, which makes them interesting thickeners. Unfortunately, the flow curves were not sensitive to the variables studied. Regarding the physical stability of the samples, interestingly, T50-6000 rpm and T25-6500 rpm exhibited the highest physical stability. Nevertheless, all samples studied exhibited good stability since, after two months of aging, the changes observed in profiles of both T% and ΔBS % were lower than 5%. In short, the stable flaxseed fiber gels obtained have a wide diversity of functional properties of interest in the food field, including stabilization and thickening, as well as the ability to slightly modify the rheological properties, provided that they are obtained under controlled processing conditions.

Overall, the application of shear by the rotor-stator homogenizer, although it decreases the stabilizer and thickening effect, could support the application of flaxseed fiber, a plant-based seed gum, in the food industry. Nevertheless, further work is needed to study the effect of very high shear, as in ultrasonic homogenization or microfluidization, on the functional properties by rheological measurements, due to these techniques being widely used in recent years.

## 4. Materials and Methods

Aqueous dispersions of flaxseed fiber (HiFood, Parma, Italy) of 2.6 wt% concentration were prepared using 1 wt% potassium sorbate (Sigma-Aldrich, Madrid, Spain) as a preservative. According to the information provided by the manufacturer, this fiber was obtained following an extraction process developed under proprietary technology (HI-FOOD R&D Dept., Parma, Italy). The molecular weight is 1.47 kD. Its chemical composition consists of 76 wt% of fiber, 9 wt% of carbohydrates, and 4 wt% of proteins, and the difference up to 100% corresponds to lipids, moisture, and ashes. It contains mainly soluble polysaccharides whose main polysaccharides are neutral highly branched arabinoxylan, principally xylose, arabinose, and galactose [49].

Two different rotor-stator devices (Ultraturrax T50 with an S50NG45F dispersion unit and Ultraturrax T25 with an S25N18GST dispersion unit, Breisgau, Germany) were used for dispersion preparation. A total of 200 g or 50 g of samples, depending on whether the equipment used was T50 or T25, respectively, was produced by adding the appropriate amount of fiber to the aqueous phase (deionized water and potassium sorbate), both at 25 °C, and then homogenized for 120 s at 2000, 4000, 6000, or 8000 rpm using Ultraturrax T50 and at 6500 or 9500 rpm using Ultraturrax T25. Subsequently, the samples were stored in a refrigerator where they remained until characterization. The amperage was measured during the preparation process to determine the power consumption.

Rheological measurements were carried out 24 h after preparation using a controlled-stress rheometer AR2000 (TA Instruments, New Castle, DE, USA) and a rough plate-plate geometry of 40 mm (PP40R, gap = 1 mm). Stress sweeps were obtained from 0.05 to 100 Pa at a fixed frequency of 1 Hz to know the extension of the linear viscoelastic region (LVR). Afterwards, frequency sweeps were carried out from 10 to 0.01 Hz at fixed stress located within the LVR. Flow curves were obtained in a range of 1 to 100 Pa employing a controlled-stress rheometer Haake Mars 40 (Thermo Fisher Scientific, Waltham, MA, USA). An equilibration time previous to the rheological tests of 3 min was used. All measurements were made in duplicate, and the values shown are the average of the two replicates performed at 20 °C.

The microstructure of the samples was observed by means of an optical microscope Axioscope A1 (Zeiss, Madrid, España) in bright field mode and different objectives such as 40× or 100×.

The physical stability of samples stored at 5 °C was determined by means of the multiple light scattering technique using Turbiscan Lab Expert (Formulation, Toulouse, France) equipment. The results are presented in the form of transmittance percentage curves (T%) and delta backscattering percentage (ΔBS %, difference between backscattering at time t and initial backscattering) as a function of storage time and the height of the measuring cell containing the sample. The Turbiscan Stability Index (TSI) was used for comparison and the estimation of the stability of aqueous dispersions. The value of this parameter was calculated from the Turbiscan device software (TurbiSoft-2.0.0.19) using the following equation:

(3) $$TSI=\frac{\sum_{h}\left|{scan}_{i\;}\left(h\right)-{scan}_{i}-1(h)\right|}{H}$$

where *scan_i_* is the backscattering for each measurement time (*i*) at a height *h*, *scan*_*i*−1_ is the backscattering for the measurement time (*i* − 1) at the same height, *h*, and *H* is the number of scans carried out on the sample.

## Figures and Tables

**Table 1:** Energy consumption per unit mass (Ev) as a function of the homogenization rate of all samples processed using a rotor-stator device.

Rotor-Stator Device	Homogenization Rate (rpm)	E (J/g)
Ultraturrax T50	2000	46.2
Ultraturrax T50	4000	62.7
Ultraturrax T50	6000	84.4
Ultraturrax T50	8000	99.0
Ultraturrax T25	6500	124.1
Ultraturrax T25	9500	132.0

**Table 2:** Fitting parameters for the Carreau model for the shear rate dependence of steady shear apparent viscosity values for the studied samples. T = 20 °C. Shear rate range: 10^−4^–2000 s^−1^.

	η_0_ (Pa·s)	SD * η_0_ (Pa·s)	γ_c_ (s^−1^)	SD * γ_c_ (s^−1^)	n	SD * n	R^2^
**T50-2000 rpm**	4103.9	126.03	0.0038	4.5·10^−4^	0.14	-	0.99
**T50-4000 rpm**	3689.7	85.011	0.0035	3.0·10^−4^	0.12	-	0.99
**T50-6000 rpm**	3952.7	53.004	0.0031	1.5·10^−4^	0.12	-	0.99
**T50-8000 rpm**	7180.5	97.057	0.0018	9.2·10^−5^	0.13	-	0.99
**T25-6500 rpm**	7397.6	67.332	0.0016	5.3·10^−5^	0.15	-	0.99
**T25-9500 rpm**	3521.2	14.639	0.0029	4.6·10^−5^	0.13	-	0.99

SD stands for the standard deviation.

## Data Availability

The data presented in this study are available on request from the corresponding author.
